# Risk factors for hypokalemia and its association with postoperative recovery in patients scheduled for radical gastrectomy: a retrospective study

**DOI:** 10.1186/s12871-023-02246-2

**Published:** 2023-08-22

**Authors:** Min Yang, Qian Li, Yan Zhou, Yun-Qing Zhu, Yu-Xuan Cui, Yu Chen, Xiao-Kai Zhou, Ming-Feng He

**Affiliations:** 1 Department of Anesthesiology and Surgery, The First Affiliated Hospital with Nanjing Medical University, Nanjing Jiangsu, Jiangsu, 210029 China; 2 The First Clinical Medical College, Nanjing Medical University, Nanjing Jiangsu, 210006 China; 3 Department of Anesthesiology and Perioperative Medicine, The First Affiliated Hospital with Nanjing Medical University, Guangzhou Road 300, Nanjing Jiangsu, 210029 China

**Keywords:** Radical gastrectomy, Hypokalemia, Risk factors, Postoperative recovery

## Abstract

**Background:**

Hypokalemia is common in patients of various operations, especially gastrointestinal surgery, which seriously affects the safety and enhanced recovery after surgery. Our study aims to explore the risk factors of preoperative hypokalemia of radical gastrectomy for gastric cancer and analyze its impact on postoperative recovery.

**Methods:**

A total of 122 patients scheduled for radical gastrectomy from September, 2022 to December, 2022 were retrospectively analyzed. According to the serum potassium level before skin incision, patients were divided into hypokalemia group (n = 64) and normokalemia group (n = 58). Factors including age, gender, BMI, ASA classification, glutamic pyruvic transaminase (ALT), glutamic oxaloacetic transaminase (AST), creatinine, blood urea nitrogen (BUN), albumin, hypertension history, whether taking calcium channel blockers, β-receptor blockers, angiotensin converting enzyme inhibitors (ACEI) or angiotensin receptor antagonist (ARB), thiazide diuretics and other drugs, anemia history, diabetes mellitus history, inability to eat or intestinal obstruction, vomiting, diarrhea, hypokalemia on admission and whether under cooperation with clinical nurse specialist were compared between groups. Univariate logistic regression analysis was used to determine risk factors for hypokalemia with *p* < 0.2 included as a cutoff. Multivariate logistic regression was used to analyze the influencing factors of preoperative hypokalemia for the indicators with differences. A receiver operating characteristic (ROC) curve was used to evaluate the efficacy of the regression model. Primary exhaust time and defecation time after surgery were compared between the two groups.

**Results:**

The use of ACEI or ARB [OR 0.08, 95% *CI* (0.01 to 0.58), *p* = 0.012] and thiazide diuretics [OR 8.31, 95% *CI* (1.31 to 52.68), *p* = 0.025], inability to eat for more than 3 days or intestinal obstruction [OR 17.96, 95% *CI* (2.16 to 149.43), *p* = 0.008], diarrhea for more than 48 h [OR 6.21, 95% *CI* (1.18 to 32.61), *p* = 0.031] and hypokalemia on admission [OR 8.97, 95% *CI* (1.05 to 77.04), *p* = 0.046] were independent influencing factors of hypokalemia before skin incision. Primary postoperative exhaust time and defecation time was significantly longer in the hypokalemia group than in the normokalemia group, no matter after laparoscopic radical gastrectomy (*p =* 0.044, *p =* 0.045, respectively) or open radical gastrectomy (*p =* 0.033, *p =* 0.019, respectively).

**Conclusion:**

Early attention and management of serum potassium in patients undergoing radical gastrectomy can better reduce perioperative adverse reactions and promote recovery of gastrointestinal function.

## Introduction

The incidence of hypokalemia during hospitalization is approximately 21.7%~23.2% [1]. As an important ion to maintain the cell membrane potential energy, abnormal potassium can lead to electrophysiological disturbances and promote the occurrence of related complications. Some studies have proposed that hypokalemia is an independent risk factor for 30-day mortality and malignant cardiac events after non-cardiac surgery, which can affect the recovery of postoperative gastrointestinal function, prolong the length of hospital stay and increase the mortality [2, 3].

Although medical practitioners routinely monitor patients’ electrolyte levels and supply in time during clinical diagnosis and treatment, but nearly half of the patients scheduled for gastrointestinal surgery still have varying degrees of hypokalemia [4]. There are few studies on the risk factors of hypokalemia, especially hypokalemia before skin incision with normokalemia after admission. In this study, we intend to elaborate and analyze the risk factors of hypokalemia before radical gastrectomy and evaluate the effect of them through a retrospective analysis. This study also reviews the impact of hypokalemia on the recovery of postoperative gastrointestinal function.

## Methods

### General information

From September 1, 2022 to December 31, 2022, patients of ASA I ~ III, aged 18 ~ 80 years, who scheduled for radical gastrectomy under general anesthesia in the Department of Gastrointestinal Surgery, the First Affiliated Hospital of Nanjing Medical University, were considered for inclusion. Patients with renal insufficiency before surgery, a history of family hypokalemia, the inability to obtain preoperative electrolyte measurements or blood gas analysis before entering the operating room and ASA IV were excluded.

The study was approved by the Ethics Committee of the First Affiliated Hospital with Nanjing Medical University (grant number 2021-SR-089, on December 12,2020) and was carried out in compliance with the Helsinki Declaration. Due to the nature of this retrospective study and the preserved anonymity of patients, a waiver of informed consent was obtained from the Ethics Committee of the First Affiliated Hospital with Nanjing Medical University.

### Research methods

#### Sample size calculation

PASS 15.0 software (NCSS, Kaysville, USA) was used for sample size calculation. As there was no related reference about the impact factor on hypokalemia in patients scheduled for radical gastrectomy, a preliminary analysis was conducted before the formal research. A logistic regression of a binary response variable (Y) on a binary independent variable (X) with a sample size of 85 observations (of which 89% are in the group X = 0 and 11% are in the group X = 1) achieves 90% power at a 0.05 significance level to detect a change in Prob from the baseline value of 0.470 to 0.930. This change corresponds to an odds ratio of 15. Considering a 15% loss of data, the total sample size was 100 at least.

#### Criteria of hypokalemia

In agreement with previous studies, a serum potassium level between 3.5 and 5.5 mmol/L was defined as normal, with levels between 3.0 and 3.5 mmol/L, 2.5 ~ 3.0 mmol/L and < 2.5 mmol/L considered as slight, moderate and severe level of hypokalemia.

#### Grouping and data collection

According to the serum potassium level before skin incision, patients were divided into hypokalemia group (Group H, serum potassium < 3.5 mmol/L) and normokalemia group (Group N, normal serum potassium) [5]. All hypokalemic patients after admission have been treated and serum potassium tested daily until it became normal. Then those patients would receive preoperative preparation normally. The patients’ characteristics such as age, gender, BMI, ASA classification, hypertension, hypotensor, anemia, diabetes mellitus, inability to eat or intestinal obstruction, vomiting, diarrhea, hypokalemia on admission and whether under cooperation with clinical nurse specialist of gastrosurgery in both groups were recorded to screen the independent risk factors of hypokalemia before skin incision. The time of postoperative primary exhaust time and defecation time were recorded, and the effects of hypokalemia on the primary time under different types of surgery were respectively compared.

#### Outcomes

The primary endpoint was to assess the relationship between hypokalemia before skin incision and patients’ characteristics, such as age, gender, BMI, ASA classification, hypertension, hypotensor, anemia, diabetes mellitus, inability to eat or intestinal obstruction, vomiting, diarrhea, hypokalemia on admission and whether under cooperation with clinical nurse specialist of gastrosurgery. The secondary endpoint was to compare the effects of hypokalemia on postoperative primary exhaust time and defecation time under different types of surgery respectively.

### Statistical analysis

SPSS 19.0 (SPSS, Inc., IBM corporation, USA) software was used for statistical analysis. R 3.3.2 (R, Lucent, USA) software was used for model validity verification.

#### Quantitative data

The Shapiro-Wilk test was used to test the normality of quantitative data. Serum potassium data with a normal distribution was reported as the mean ± standard deviation (‾*x ± sd*) and compared between groups using the paired-sample t-test. For patients’ characteristic data, the independent two sample data was reported as the mean ± standard deviation (‾*x ± sd*) and compared between groups using the independent two sample t-test.

#### Qualitative data

The categorical data was analyzed in terms of number of cases and percentages (%), and comparison between groups was performed by chi-square test or Fisher’s exact tests.

#### Logistic regression analysis

After confirming residual independence and the logit (P) transformation of the independent variable follows a linear relationship with the dependent variable, a univariate logistic regression analysis was used to determine risk factors for hypokalemia. The independent variables in the univariate model with *p* < 0.2 were included as covariates in a multivariate logistic regression analysis, which was used to identify risk factors for hypokalemia before entering the operating room. The receiver operating characteristic (ROC) curve was used to evaluate the effect of the risk factors on hypokalemia. *P* < 0.05 was considered statistically significant.

## Results

### Enrollment of patients

**Fig. 1:**
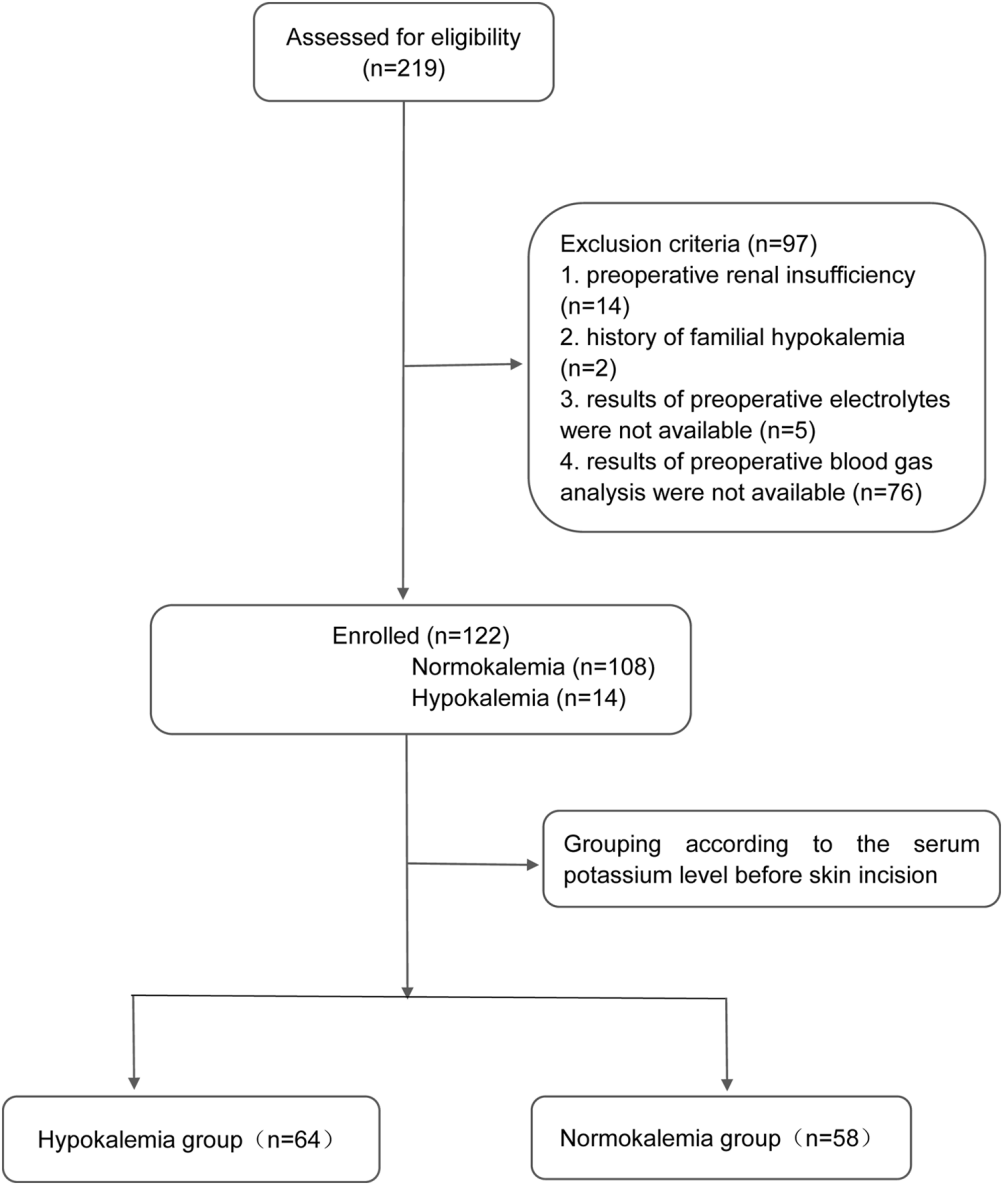
Flowchart of enrollment

The final analysis was based on the data of 122 patients who were able to obtain serum electrolyte on admission and serum potassium levels measured by arterial blood gas analysis after entering the operating room. Detailed inclusion and exclusion procedures are shown in Fig. 1. Among these patients, 108 (88.5%) patients’ potassium were normal and 14 (11.5%) were hypokalemic.

### Serum potassium of patients with normal serum potassium on admission when entered the operating room

All patients enrolled were able to obtain their serum potassium after admission, before skin incision and before discharge. In patients with normal serum potassium on admission, the serum potassium was 3.9 ± 0.2 mmol/L after admission, 3.5 ± 0.3 mmol/L before skin incision, and 4.0 ± 0.4 mmol/L before discharge. The serum potassium after admission and before discharge were significantly higher than those before skin incision [paired-*t* value 12.45, 95% *CI* 0.42 (0.36 to 0.49), *p* < 0.017; paired-*t* value 12.90, 95% *CI* -0.51 (-0.59 to -0.43), *p* < 0.017, respectively], but there was no significant difference between the serum potassium after admission and before discharge [paired-*t* value − 1.91, 95% *CI* -0.08 (-0.16 to 0.00), *p* > 0.017] (Table 1).

**Table 1:** Serum potassium of patients with normal serum potassium on admission when entered the operating room

Time	Serum potassium (mmol/L)	Paired sample	Difference and 95% *CI* (mmol/L)	*Paired-t* test	
				*t* value	*p* value
After admission	3.9 ± 0.2	After admission- before skin incision	0.42(0.36 to 0.49)	12.45	< 0.001
Before skin incision	3.5 ± 0.3	After admission - before discharge	-0.08(-0.16 to 0.00)	-1.97	0.051
Before discharge	4.0 ± 0.4	Before skin incision - before discharge	-0.51(-0.59 to -0.43)	12.90	< 0.001

### Univariate logistic regression analysis of hypokalemia

Univariate logistic regression analysis of the association between perioperative characteristics and the risk of developing hypokalemia was carried. A cutoff *p* < 0.2 was used as a filter for determining whether the independent variable was an appropriate factor for the forward multivariate analysis. Univariate logistic regression analysis showed that the two groups of patients had statistically significant differences in glutamic pyruvic transaminase (ALT), blood urea nitrogen (BUN), the use of angiotensin converting enzyme inhibitors (ACEI) or angiotensin receptor antagonist (ARB), the use of thiazide diuretics, inability to eat or intestinal obstruction, vomiting, diarrhea, hypokalemia on admission (*p* < 0.2) (Table 2).

**Table 2:** Univariate logistic regression analysis of influencing factors of preoperative hypokalemia in gastrointestinal surgery

	Group H(n = 64)	Group N (n = 58)	*OR* value (95% *CI*)	*p* value
Age(y)	63.33±11.72	62.28±11.12	1.01(0.98 to 1.04)	0.610
Gender[n(%)] Male Female	64 44(68.8%) 20(31.2%)	58 45(77.6%) 13(22.4%)	0.64(0.28 to 1.43) 1.00	0.274
BMI(kg/m2)	23.12±2.78	23.55±3.78	0.98(0.86 to 1.07)	0.476
ASA classification [n(%)]				
I	3(4.7%)	1(1.7%)	1.00	
II	50(78.1%)	50(86.2%)	0.33(0.03 to 3.31)	0.349
III	11(17.2%)	7(12.1%)	0.52(0.05 to 6.09)	0.605
AST (U/L)	22.18±13.24	22.03±9.77	1.00(0.97 to 1.03)	0.942
ALT (U/L)	17.15±11.48	20.54±13.51	0.98(0.98 to 1.00)	**0.137**
Creatinine (umol/L)	69.38±16.61	72.42±20.80	0.99(0.97 to 1.01)	0.370
BUN (mmol/L)	5.35±2.10	6.85±6.76	0.90(0.78 to 1.03)	**0.130**
Hypertension [n(%)]	12(18.8%)	13(22.4%)	0.80(0.33 to 1.93)	0.617
Calcium channel blockers [n(%)]	1(1.6%)	1(1.7%)	0.91(0.06 to 14.80)	0.944
β-receptor blockers[n(%)]	4(6.3%)	4(6.9%)	0.90(0.22 to 3.78)	0.885
ACEI or ARB[n(%)]	3(4.7%)	10(17.2%)	0.24(0.62 to 0.91)	**0.035**
Thiazide diuretics [n(%)]	7(10.9%)	2(3.4%)	3.44(0.68 to 17.27)	**0.134**
Anemia [n(%)]	17(26.6%)	18(31.0%)		
Light	15(23.4%)	14(24.1%)	0.91(0.39 to 2.11)	0.830
Moderate	1(1.6%)	1(1.7%)	0.85(0.05 to 14.05)	0.910
Severe	1(1.6%)	3(5.2%)	0.28(0.03 to 2.84)	0.283
Diabetes mellitus [n(%)]	5(7.8%)	3(5.2%)	1.55(0.35 to 6.81)	0.559
Inability to eat or Intestinal obstruction ^a^ [n(%)]	11(17.2%)	3(5.2%)	3.81(1.01 to 14.40)	**0.049**
Albumin (g/L)	37.94±7.41	39.64±8.81	0.97(0.93 to 1.02)	0.255
Vomiting ^b^ [n(%)]	11(17.2%)	5(8.6%)	2.20(0.715 to 6.77)	**0.169**
Diarrhea ^c^[n(%)]	16(25.0%)	3(5.2%)	6.11(1.68 to 22.26)	**0.006**
Hypokalemia on admission [n(%)]	13(20.3%)	1(1.7%)	14.53(1.84 to 115.00)	**0.031**
Clinical nurse specialist of gastrosurgery^d^ [n(%)]	57(89.1%)	53(93.0%)	0.768(0.230 to 2.569)	0.668

^a^ Anemia- Light, hemoglobin concentration 110 g/L ~ lower limit of normal reference value; Moderate, hemoglobin concentration 80 g/L ~ 109 g/L; Severe, hemoglobin concentration < 80 g/L;

^a^ Inability to eat for more than 3 days or have clinical or imaging signs of intestinal obstruction;

^b^ Vomiting for more than 48 h;

^c^ Diarrhea for more than 48 h

^d^Operation was under cooperation with clinical nurse specialist of gastrosurgery

Group H, Hypokalemia group; Group N, Normokalemia group;

ALT, glutamic pyruvic transaminase; AST, glutamic oxaloacetic transaminase; BUN, blood urea nitrogen; ACEI, angiotensin converting enzyme inhibitors; ARB, angiotensin receptor antagonist;

### Multivariate logistic regression analysis of hypokalemia

**Fig. 2:**
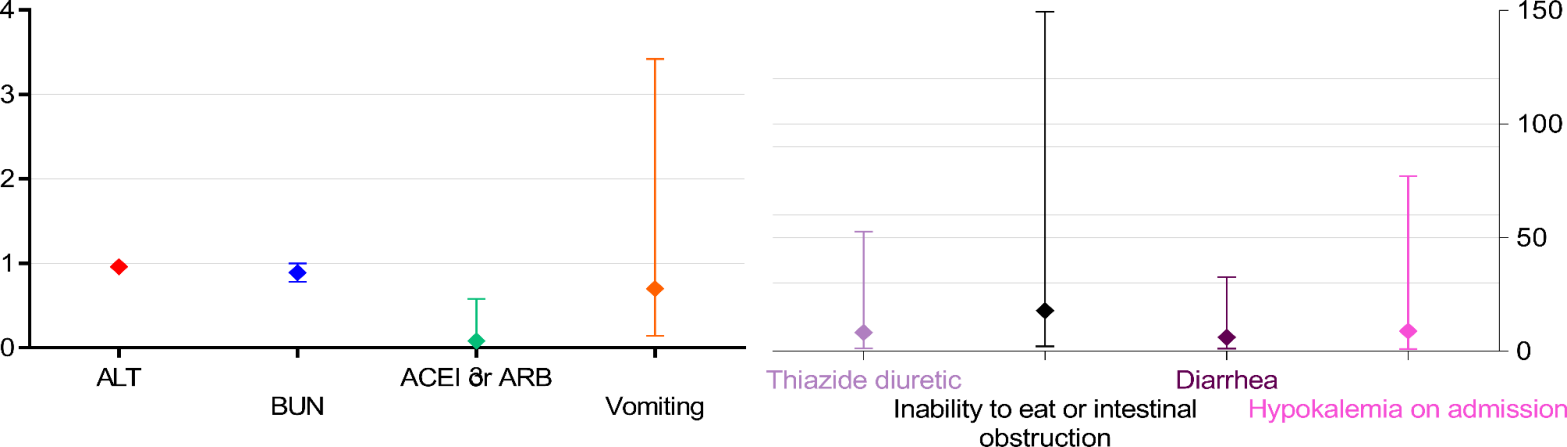
Risk factors in multivariable analyses showing adjusted odds ratio(OR) and 95% *CI* Abbreviations: ALT, glutamic pyruvic transaminase; BUN, blood urea nitrogen; ACEI, angiotensin converting enzyme inhibitors; ARB, angiotensin receptor antagonist

A multivariate binary logistic regression equation was constructed by ALT, BUN, the use of ACEI or ARB, the use of thiazide diuretic, inability to eat or intestinal obstruction, vomiting, diarrhea, and hypokalemia on admission (Table 3). The results found that the use of ACEI or ARB reduced the risk of hypokalemia with statistical significance [*OR* = 0.08, 95% *CI* (0.01 to 0.58), *p* < 0.05]; the use of thiazide diuretic increased the risk of hypokalemia with statistical significance [*OR* = 8.31, 95% *CI* (1.31 to 52.68), *p* < 0.05]; inability to eat for more than 3 days or with imaging signs of intestinal obstruction increased the risk of hypokalemia with statistical significance [*OR* = 17.96, 95% *CI* (2.16 to 149.43), *p* < 0.05]; diarrhea for more than 48 h increased the risk of hypokalemia with statistical significance [*OR* = 6.21, 95% *CI* (1.18 to 32.61), *p* < 0.05]; hypokalemia on admission increased the risk of preoperative hypokalemia with statistical significance [*OR* = 8.97, 95% *CI* (1.05 to 77.04), *p* < 0.05] (Fig. 2).

**Table 3:** Multivariate logistic regression analysis of influencing factors of preoperative hypokalemia in gastrointestinal surgery

	Grouping	*b* value	*b* value standard error	Wald $\chi 2$value	*OR* value (95% *CI*)	*p* value
ALT(U/L)		-0.04	0.02	3.52	0.96(0.93 to 1.00)	0.061
BUN(mmol/L)		-0.12	0.07	3.30	0.89(0.78 to 1.00)	0.069
ACEI or ARB[n(%)]	Group N Group H	-2.48	0.99	6.29	0.08(0.01 to 0.58)	**0.012**
Thiazide diuretic [n(%)]	Group N Group H	2.12	0.94	5.05	8.31(1.31 to 52.68)	**0.025**
Inability to eat or intestinal obstruction [n(%)]	Group N Group H	2.89	1.08	7.14	17.96(2.16 to 149.43)	**0.008**
Vomiting ^a^ [n(%)]	Group N Group H	-0.35	0.81	0.192	0.70(0.14 to 3.42)	0.662
Diarrhea^b^ [n(%)]	Group N Group H	1.83	0.85	4.65	6.21(1.18 to 32.61)	**0.031**
Hypokalemia on admission [n(%)]	Group N Group H	2.19	1.10	4.00	8.97(1.05 to 77.04)	**0.046**

^a^ Vomiting for more than 48 h;

^b^ Diarrhea for more than 48 h;

Group H, Hypokalemia group; Group N, Normokalemia group;

ALT, glutamic pyruvic transaminase; BUN, blood urea nitrogen; ACEI, angiotensin converting enzyme inhibitors; ARB, angiotensin receptor antagonist;

### Multivariate regression model

**Fig. 3:**
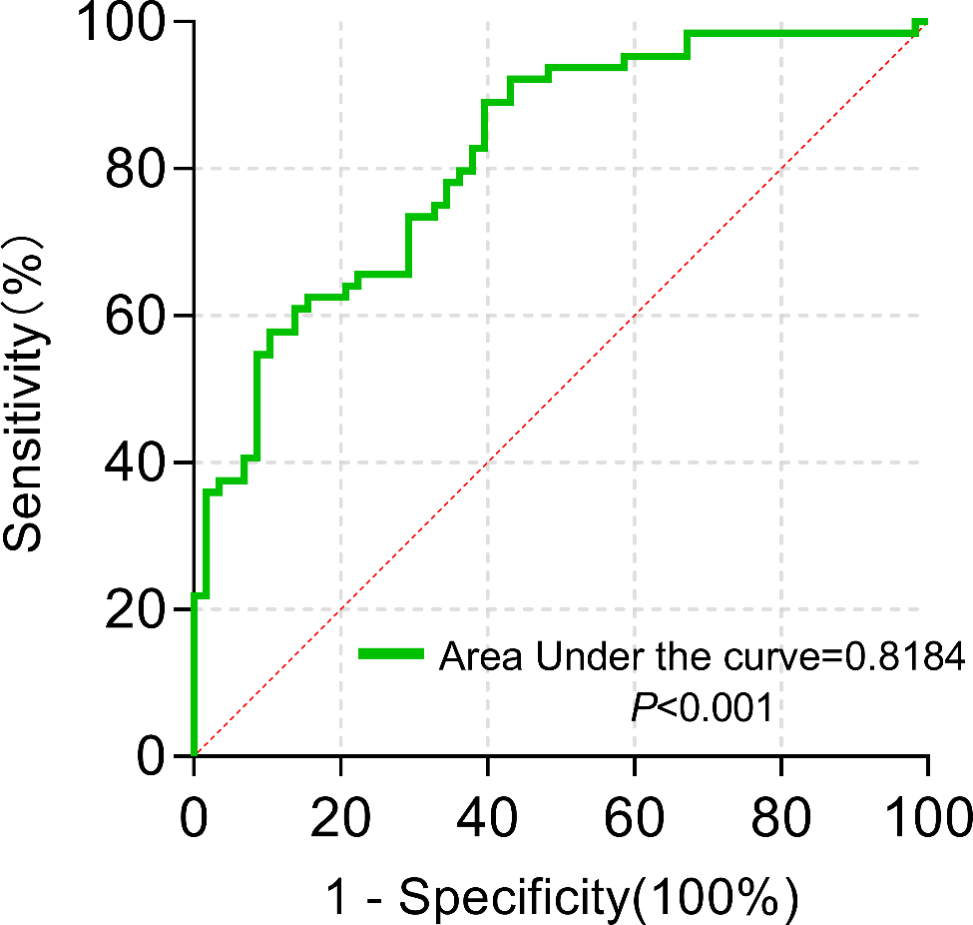
ROC curves were used to analyze multivariate model

We calculated area under ROC curve (AUC), sensitivity and specificity of the model. It suggested that AUC was 0.82 [*p* < 0.001, 95% *CI* (0.74 to 0.89)]. A probability cutoff of 0.47 was selected based on the Youden index as offering the optimal balance between sensitivity and specificity (Youden index = sensitivity plus specificity minus 1), with values above this threshold reflecting a high risk of hypokalemia. Applying this cutoff, the prediction model achieved sensitivity of 73.4% and specificity of 70.7% (Fig. 3). The − 2log-likelihood ratio, Cox and Snell R^−^square, Nagelkerke R-square, Hosmer-Lemeshow test, Akaike Information Criterion (AIC) and Bayesian Information Criterion (BIC) to evaluate the estimates of models. The − 2log-likelihood ratio, Cox and Snell R^−^square, Nagelkerke R-square, p value of Hosmer-Lemeshow test, AIC and BIC were 125.855, 0.297, 0.396, 0.722, 132.8 and 149, respectively.

### Model validity verification

**Fig. 4:**
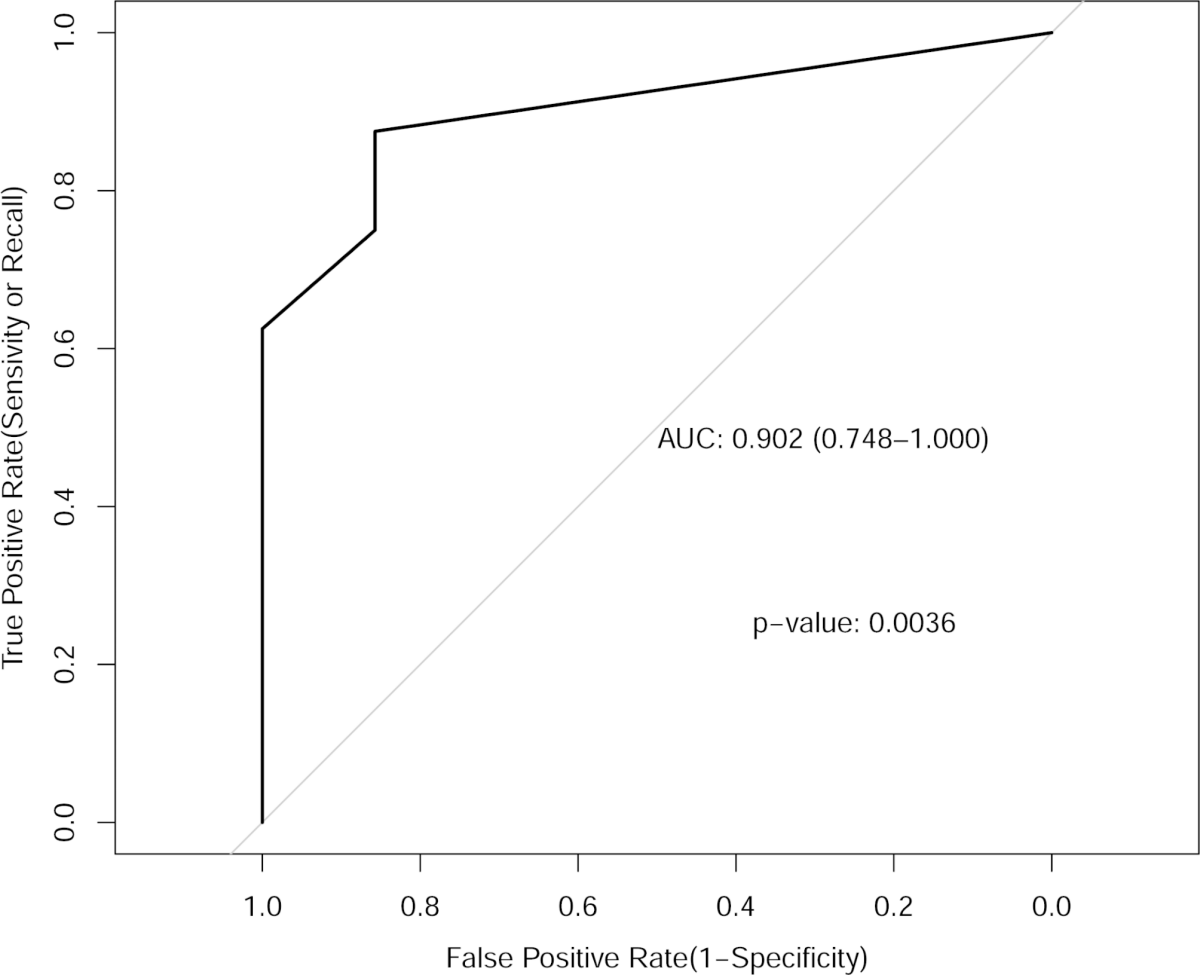
K-Folder Cross Validation was used for model validity verification

K-Folder Cross Validation was used for model validity verification. The result indicates that mean AUC was 0.902 [95% *CI* (0.748 to 1.000), p < 0.05] (Fig. 4).

### Effects of preoperative hypokalemia on postoperative recovery

Patients in the two groups were stratified according to the types of surgery, and the primary exhaust time and defecation time after laparoscopic radical gastrectomy and open gastrectomy for gastric cancer were compared respectively in the two groups. After laparoscopic radical gastrectomy, the primary exhaust time and defecation time in Group H were longer than those in Group N, and the difference was statistically significant [3.4±0.8 vs. 3.0±0.7, 95% *CI* (0.11 to 0.80), *p* < 0.05; 5.0±0.9 vs. 4.5±0.8, 95% *CI* (0.01 to 0.90), *p* < 0.05, respectively]. After open radical gastrectomy, the primary exhaust time and defecation time in Group H were longer than those in Group N, and the difference was statistically significant [4.5±0.6 vs. 4.1±0.8, 95% *CI* (0.03 to 0.77), *p* < 0.05; 5.7±0.7 vs. 5.2±0.9, 95% *CI* (0.09 to 0.92), *p* < 0.05, respectively] (Table 4).

**Table 4:** Comparison of the primary exhaust time and defecation time after different types of surgery

	Laparoscopic radical gastrectomy group(n = 61)		*t* value	*p* value	Open gastrectomy group(n = 61)		*t* value	*p* value
	Group H(n = 33)	Group N(n = 28)			Group H(n = 31)	Group N(n = 30)		
Primary exhaust time(d)	3.4±0.8	3.0±0.7	2.06	0.044	4.5±0.6	4.1±0.8	2.19	0.033
Primary defecation time(d)	5.0±0.9	4.5±0.8	2.05	0.045	5.7±0.7	5.2±0.9	2.41	0.019

Group H, Hypokalemia group; Group N, Normokalemia group;

## Discussion

Similar to the finding of Wang et al., the incidence rate of hypokalemia before skin incision was 52.5% in this study, with 11.5% patients presenting with severe hypokalemia [4]. The data of 122 patients undergoing radical gastrectomy were included for regression analysis. It was found that the risk factors of hypokalemia included the use of potassium-removing diuretics before surgery, inability to eat for more than 3 days or intestinal obstruction, diarrhea for more than 48 h and hypokalemia on admission. The use of ACEI or ARB before surgery was a protective factor for preoperative hypokalemia, while the use of other hypotensor was not associated with preoperative hypokalemia. After treatment, the patients’ serum potassium returned to normal 48 h after surgery, but preoperative hypokalemia was still associated with the primary exhaust time and defecation time.

The incidence of preoperative hypokalemia in gastrointestinal surgery is significantly higher than that of general inpatients [1]. Zhu et al. noted that the incidence of hypokalemia before pneumoperitoneum establishment was 70.37% and pointed out that hypertension and the use of two or more bowel preparation medications were risk factors for hypokalemia before pneumoperitoneum establishment [6]. Boersema et al. suggested that gastrointestinal surgery often causes electrolyte abnormalities throughout the perioperative period due to the need for gastrointestinal preparation [7]. Patients undergoing gastrointestinal surgery were at a high risk of hypokalemia, with the incidence was 49.6% in a study included 999 patients by Wang et al., in which both female and the use of laxative were independent risk factors [4].

Hypokalemia is very common in hospitalized patients. Multiple factors may contribute to hypokalemia, including insufficient intake, excessive consumption and abnormal distribution. For laparoscopic radical gastrectomy, rigorous gastrointestinal preparation may lead to hypokalemia. Polyethylene glycol is the most common bowel preparation medications. Studies have shown that hypokalemia is a common complication of bowel preparation with polyethylene glycol [8]. Patients with gastric cancer who are combined with constipation often take other laxatives such as lactulose and mannitol prior to hospitalization, which can also lead to hypokalemia [9]. Certainly, patients who take two or more laxatives are more likely to cause hypokalemia. Therefore, serum potassium should be monitored prior to gastrointestinal preparation for these patients.

In addition to the loss of potassium through the gastrointestinal tract, the use of potassium-removing diuretics can also lead to hypokalemia [10]. Thiazide diuretics, which are often taken by patients with cardiovascular disease (e.g. hypertension), can also cause hypokalemia [11]. Although there were more hypertensive patients in the hypokalemia group than in the normokalemia group, there was no statistical difference, which may be due to the insufficient sample size. However, the utilization rate of hypotensor including thiazides diuretics was significantly different between the two groups. Multivariate logistic regression analysis suggested that the use of thiazide diuretics was an independent risk factor for hypokalemia in our study. This kind of drug promotes potassium excretion by inhibiting sodium reabsorption while increasing the exchange of sodium and potassium [12]. It is noteworthy that the use of ACEI or ARB before surgery is a protective factor, and the utilization rate was significantly lower in the hypokalemic group than in the normokalemia group. Multivariate logistic regression analysis also suggested that the use of ACEI or ARB could reduce the risk of hypokalemia.

Although hypokalemia is usually asymptomatic, it remains a risk factor for gastrointestinal dysfunction and perioperative cardiac arrhythmia [13–16]. In addition, hypokalemia may also lead to delayed recovery after anesthesia, prolonged hospital stay, increased all-cause and cardiovascular-related mortality [1, 17, 18]. Our study also indicated that the primary exhaust time and defecation time was significantly delayed in patients with hypokalemia. Therefore, maintaining normal perioperative serum potassium is important in reducing gastrointestinal-related mortality and improving recovery of gastrointestinal function after surgery. In fact, ERAS also recommends prophylactic potassium supplementation [7]. Another study even suggested that the prevention of hypokalemia before admission could be effective in promoting rapid postoperative recovery after transabdominal surgery [19]. In our study, the rate of hypokalemia before gastrointestinal preparation was not high (11.5%), and these patients’ hypokalemia were all considered as slight. In contrast, the incidence of hypokalemia was 52.5% after gastrointestinal preparation and 11.5% of them were considered as moderate or severe hypokalemia. Studies suggested that early monitoring of serum potassium could lead to earlier correction of hypokalemia and more effective surgical recovery [19, 20]. Therefore, early potassium supplementation under close supervision is highly recommended for patients scheduled for gastrointestinal surgery especially after gastrointestinal preparation. It requires vigilance for the occurrence of hypokalemia. Hypertension was not an independent risk factor for hypokalemia in our study, but whether hypertensive patients are more likely to develop hypokalemia during gastrointestinal preparation may require more studies to confirm.

There are several limitations of the present study which need to be declared. First, the sample size of this study was limited, so a larger, multicenter, evidence-based medical study is clinically needed. Second, this study did not include other detailed information, such as the patients’ nutritional status and bowel preparation modalities. Third, the primary exhaust time and defecation time are the references for clinical evaluation of gastrointestinal function, and subsequent studies can accurately evaluate the recovery of gastrointestinal function by nuclide recording or other methods.

## Conclusions

In conclusion, the use of thiazide diuretic, inability to eat or intestinal obstruction, diarrhea and hypokalemia on admission are independent risk factors for hypokalemia before radical gastrectomy for gastric cancer, while the use of ACEI or ARB is a protective factor. The incidence of hypokalemia, even severe hypokalemia, before radical gastrectomy is high. Hypokalemia can lead to prolonged primary defecation time after surgery and affect the early recovery. Therefore, we believe that early monitoring and management of patients at high risk of hypokalemia can better reduce perioperative adverse reactions and promote early recovery of gastrointestinal function.

## Data Availability

We declared that materials described in the manuscript, including all relevant raw data, will be freely available to any scientist wishing to use them for non-commercial purposes, without breaching participant confidentiality. The data could be obtained from the corresponding author upon reasonable request from the publication date.

## Abbreviations

ALT: Glutamic pyruvic transaminase
AST: Glutamic oxaloacetic transaminase;
BUN: Blood urea nitrogen;
ACEI: Angiotensin converting enzyme inhibitors;
ARB: Angiotensin receptor antagonist;
ROC: Receiver operating characteristic.
AUC: Area under receiver operating characteristic curve.
